# Delivery of Molecules into Human Corneal Endothelial Cells by Carbon Nanoparticles Activated by Femtosecond Laser

**DOI:** 10.1371/journal.pone.0132023

**Published:** 2015-07-02

**Authors:** Clotilde Jumelle, Cyril Mauclair, Julien Houzet, Aurélien Bernard, Zhiguo He, Fabien Forest, Michel Peoc’h, Sophie Acquart, Philippe Gain, Gilles Thuret

**Affiliations:** 1 Corneal Graft Biology, Engineering and Imaging Laboratory, EA2521, SFR143, Faculty of Medicine, Saint-Etienne, France; 2 Hubert Curien Laboratory, UMR-CNRS 5516, Pôle Optique Rhône-Alpes, Saint-Etienne, France; 3 GIE Manutech-Ultrafast Surfacing Design, Saint-Etienne, France; 4 Department of Pathology, University Hospital, Saint-Etienne, France; 5 Department of Ophthalmology, University Hospital, Saint-Etienne, France; 6 Eye Bank, Etablissement Français du Sang Loire/Auvergne, Saint-Etienne, France; 7 Institut Universitaire de France, Paris, France; University of Florida, UNITED STATES

## Abstract

Corneal endothelial cells (CECs) form a monolayer at the innermost face of the cornea and are the engine of corneal transparency. Nevertheless, they are a vulnerable population incapable of regeneration in humans, and their diseases are responsible for one third of corneal grafts performed worldwide. Donor corneas are stored in eye banks for security and quality controls, then delivered to surgeons. This period could allow specific interventions to modify the characteristics of CECs in order to increase their proliferative capacity, increase their resistance to apoptosis, or release immunosuppressive molecules. Delivery of molecules specifically into CECs during storage would therefore open up new therapeutic perspectives. For clinical applications, physical methods have a more favorable individual and general benefit/risk ratio than most biological vectors, but are often less efficient. The delivery of molecules into cells by carbon nanoparticles activated by femtosecond laser pulses is a promising recent technique developed on non-adherent cells. The nanoparticles are partly consummated by the reaction releasing CO and H_2_ gas bubbles responsible for the shockwave at the origin of cell transient permeation. Our aim was to develop an experimental setting to deliver a small molecule (calcein) into the monolayer of adherent CECs. We confirmed that increased laser fluence and time exposure increased uptake efficiency while keeping cell mortality below 5%. We optimized the area covered by the laser beam by using a motorized stage allowing homogeneous scanning of the cell culture surface using a spiral path. Calcein uptake reached median efficiency of 54.5% (range 50.3–57.3) of CECs with low mortality (0.5%, range (0.55–1.0)). After sorting by flow cytometry, CECs having uptaken calcein remained viable and presented normal morphological characteristics. Delivery of molecules into CECs by carbon nanoparticles activated by femtosecond laser could prove useful for future cell or tissue therapy.

## Introduction

Corneal endothelial cells (CECs) are the sole engine for corneal transparency but cannot divide in vivo in humans and are therefore vulnerable. They form a monolayer of tightly packed cells in the posterior part of the cornea, separating it from the aqueous humor. Equipped with numerous ionic pumps of different and complementary functions and with a rich mitochondrial network, they permanently maintain the gradient of concentration of ionic species between the corneal stroma and aqueous humor, which extract water from the hydrophilic stroma. This active deswelling maintains the organization of collagen fibers implicated in crystal-clear corneal transparency. This population of approximately 350000 CECs in each cornea is therefore paramount in visual function, but several diseases specifically target CECs, resulting in permanent corneal edema. Up to now, the only option for treating corneal edema is corneal grafting using a donor cornea. Corneal endothelial diseases are responsible for one third of corneal grafts performed worldwide. There is a great global scarcity of corneal tissue. We recently identified 185000 grafts performed annually in the world (personal communication) whereas several million would be necessary to eradicate the 5 million cases of bilateral corneal blindness [1] and 10–12 million unilateral cases [2]. Alternative solutions are therefore crucial. Among possibilities, is improving the endothelial quality of stored corneas to reduce the number of tissues discarded at the end of storage period. By increasing endothelial cell density (ECD) during storage period, an estimated 20–30% of usually discarded grafts could be spared. In addition, corneas with super-high ECD would survive longer in recipients, thus avoiding or postponing regrafting which, in very active centers, may be one of the three main indications for corneal grafting. Increasing ECD seems possible by interfering with cell cycle regulation [3]. Complementarily, production by CECs of anti-apoptotic molecules like p35 of Bcl-x(L) would reduce ECD attrition during storage [4] and probably also during and post operation. Another solution to prolong graft survival in recipients is to reduce the number of immune rejection episodes. Repeated rejections, particularly the most severe ones with delayed treatment, decrease ECD and result in chronic endothelial dysfunction that requires regrafting in a difficult situation of higher failure risk due to allosensitization. Gene therapy of CECs, allowing a release of immunosuppressive molecules like IL10, would reduce rejection episode frequency and severity [5]. All these potential interventions require pre-graft altering of CECs with minimal toxicity. Fortunately, the cornea can be stored in eye banks for several weeks using organ culture, the preferred method in Europe [6]. This long period in conditions that favor metabolic activity (unlike short-term cold storage) provides an exceptional opportunity to alter CECs while controlling the efficiency and safety of the intervention, with a high level of safety before grafting the modified cornea in the recipient.

Numerous methods have previously been experimentally assessed to deliver molecules into CECs. Overall, viral vectors are the most efficient methods for delivering nucleic acids into cells, and CECs are no exception [7–10]. Nevertheless, for clinical applications in this field, an important issue is method acceptance by the health authorities. For instance, gene therapy using viruses, which can induce endogenous recombination, oncogenic effects and immunological stimulation, would unlikely be accepted for non-lethal diseases, nor for visual disability less severe than blindness caused by diffuse retinal or optic nerve diseases. Additionally, unlike transfection of cells for experimental purposes where high mortality may be tolerable, delivery of molecules into CECs for therapeutic purpose requires minimal collateral damage. It also seems unrealistic to remove the 350000 CECs from the cornea, treat them in suspension, then reseed them in their initial location, therefore only in situ delivery methods on the intact endothelial monolayer would be a useful approach.

Among non-viral methods, various categories of chemical vectors have been developed (phospholipids, polymers, chitosan) and some have been applied to CECs [11–13]. They have similar modes of action, based on natural pathways of molecule internalization by cells, like endocytosis. Mostly used for gene delivery, they usually have low toxicity but also low efficiency compared to viruses, because of the high risk of nucleic acid degradation during internalization.

**Fig 1:**
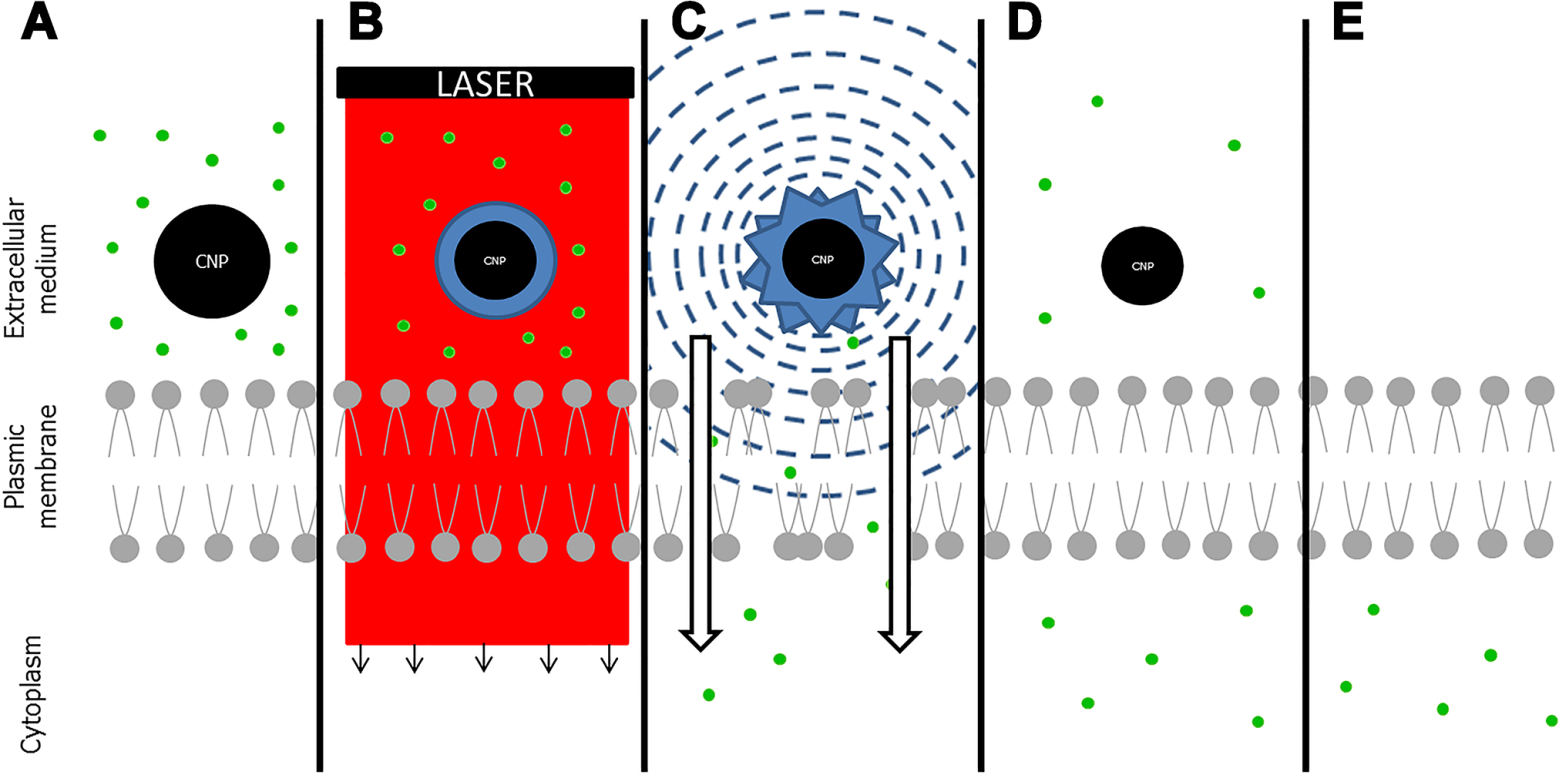
Principle of delivery of molecules by carbon nanoparticles activated by femtosecond laser pulses (adapted from Chakravarty et al. [21]). (A) Cells are immersed in a solution of carbon nanoparticles (CNPs) and molecules of interest; (B) a laser pulse triggers CNP heating, C(s)+H_2_O(l) = CO(g)+H_2_(g) (s: solid; l: liquid; g: gas) and gas bubble formation. (C) once laser stops, bubbles collapse, resulting in shockwave propagation to cells. Cell membranes are permeabilized and the molecules penetrate into the cells; (D) cell membranes quickly return to their normal organization, limiting mortality; (E) rinsing removes the CNPs not consumed during reaction and molecules of interest that did not penetrate into cells.

Physical methods are the last category used to deliver molecules directly and specifically into target cells. They work by transiently destabilizing the phospholipid bilayer cell membrane to allow therapeutic molecules crossing of the impermeable barrier. Several physical principles have been used in numerous cell types. Ballistic: the gene gun propels gold nanoparticles conjugated with genes at high velocity directly into cells. This may be efficient but is difficult to control and showed high damage to cells, especially CECs [14]. Electricity: electroporation by very short high voltage pulses triggers the formation of holes in cell membranes and electrophoresis of charged molecules. We previously reported 7 ± 11% of CECs transfected by GFP genes in situ in stored human corneas [15]. Iontophoresis uses low voltage current to push charged molecules across cell membranes, but is limited by the size of molecules delivered, precluding gene delivery in particular [16]. Both electrical methods have been combined to deliver siRNA into mice corneal epithelial cells [17]. Ultrasound: sonoporation uses ultrasound waves to disrupt cell membranes thanks to contrast agents such as microbubbles that collapse and create membrane pores. This has already allowed in vitro and in vivo transfection of rabbit corneal keratocytes [18]. Light: photoporation uses lasers to permeabilize cell membranes, with two principles described for gene therapy. Optoinjection by focusing a laser beam onto the cell surface to create a pore is an efficient single-cell treatment but remains of limited use for application to whole tissues. Transfection by laser-induced stress waves uses short laser pulses to generate stress waves that induce a transient increase of cell permeability without affecting cell viability [19]. However, none of these photoporation techniques has been tested on ocular tissues. Recently, nanoparticles (i.e. between 1 and 100 nanometers in size) activated by femtosecond laser (FsL) pulses have been used to deliver molecules of a wide range of sizes into cells with very high efficiency and without significant toxicity. Plasmonic effect induced by gold nanoparticles activated by FsL pulses has been used to transfect plasmids into non-confluent adherent melanoma cells [20], and photoacoustic effect of carbon nanoparticles (CNPs) activated by FsL (Fig 1) has been used to deliver calcein, albumin and plasmid into prostate cancer cells in suspension [21].

In the present article, we describe for the first time the delivery of a small molecule (calcein) into a fully confluent adherent monolayer of CECs, mimicking the human corneal endothelium, using carbon nanoparticles activated by FsL pulses.

## Materials and Methods

### Materials

Immortalized CEC line B4G12 (ACC 647) was purchased from DSMZ (Braunschweig, Germany). Human Endothelial Serum-Free Medium (SFM), 24 well tissue culture plates, ZO-1 antibodies and AlexaFluor 488 Goat anti-mouse IgG (H+L) antibodies were purchased from Fisher Bioblock Scientific (Vaulx-Milieu, France). Fibroblast Growth Factor (FGF-2), calcein, sodium dodecyl sulfate (SDS), propidium iodide (PI), trypsin/EDTA 1X and Hoechst 33342 were purchased from Sigma-Aldrich (Saint-Quentin Fallavier, France). Balanced Salt Solution (BSS) was purchased from Alcon (Rueil-Malmaison, France). Black carbon nanoparticles of 15-nanometer diameter (Black Pearls 470) were purchased from Cabot Corp and Business (Billerica, MA). Ti:Sa laser system was purchased from Thalès (Neuilly-sur-Seine, France). ANT-130 XYZ motorized stage and A3200 Software-Based Machine Controller were purchased from Aerotech (Pittsburgh, PA). The inverted fluorescence microscope was purchased from Olympus (Tokyo, Japan). FACSCalibur flow cytometer was purchased from Becton Dickinson (Franklin Lakes, NJ). Gentamycin was purchased from PAA Laboratories, (Pasching, Austria). Phalloidin was purchased from Cytoskeleton (Denver, CO).

### Human Corneal Endothelial Cells

The immortalized CEC line B4G12 was cultivated in Human-Endothelial-SFM supplemented by 10 ng/mL FGF-2 at 37°C in a humidified atmosphere with 5% CO_2_. In order to obtain a cell monolayer with an area close to the actual human corneal endothelium area, CECs were seeded in 24-well plates. The well diameter of 15.6 mm, larger than corneal diameter, prevented artifacts caused by the vertical well walls during laser exposure. To ensure reproducible ECD, 1x10^4^ cells were seeded in each well and cultivated for 10 days, corresponding to a tightly packed cell monolayer similar to the native corneal endothelium.

### Nanoparticles and irradiation medium

Stock CNP suspension solution was prepared by sonication of 0.03g of carbon nanoparticles in 50mL of BSS with 0.077g SDS. This solution was autoclaved as per the three phases recommended for liquids: air evacuation, sterilization at 121°C for 20 min (norm NFS 90–320), and natural cooling. This stock solution was stored at room temperature until use. Stock solution of 90μg/mL of calcein was prepared by dissolution in BSS and sonication for 2 minutes. Just before the experiments, an intermediate CNP solution was prepared with 5 mL of stock CNP solution and 28.5 mL of BSS, and a solution containing CNPs, calcein and CEC culture medium 1:1:1 v/v/v was prepared. Final concentration of calcein was 10 μg/mL in the irradiation medium. Three concentrations of CNPs were first assessed (10, 30 and 90 μg/mL), and 30 μg/mL finally used for all the subsequent experiments.

### Femtosecond laser irradiation

**Fig 2:**
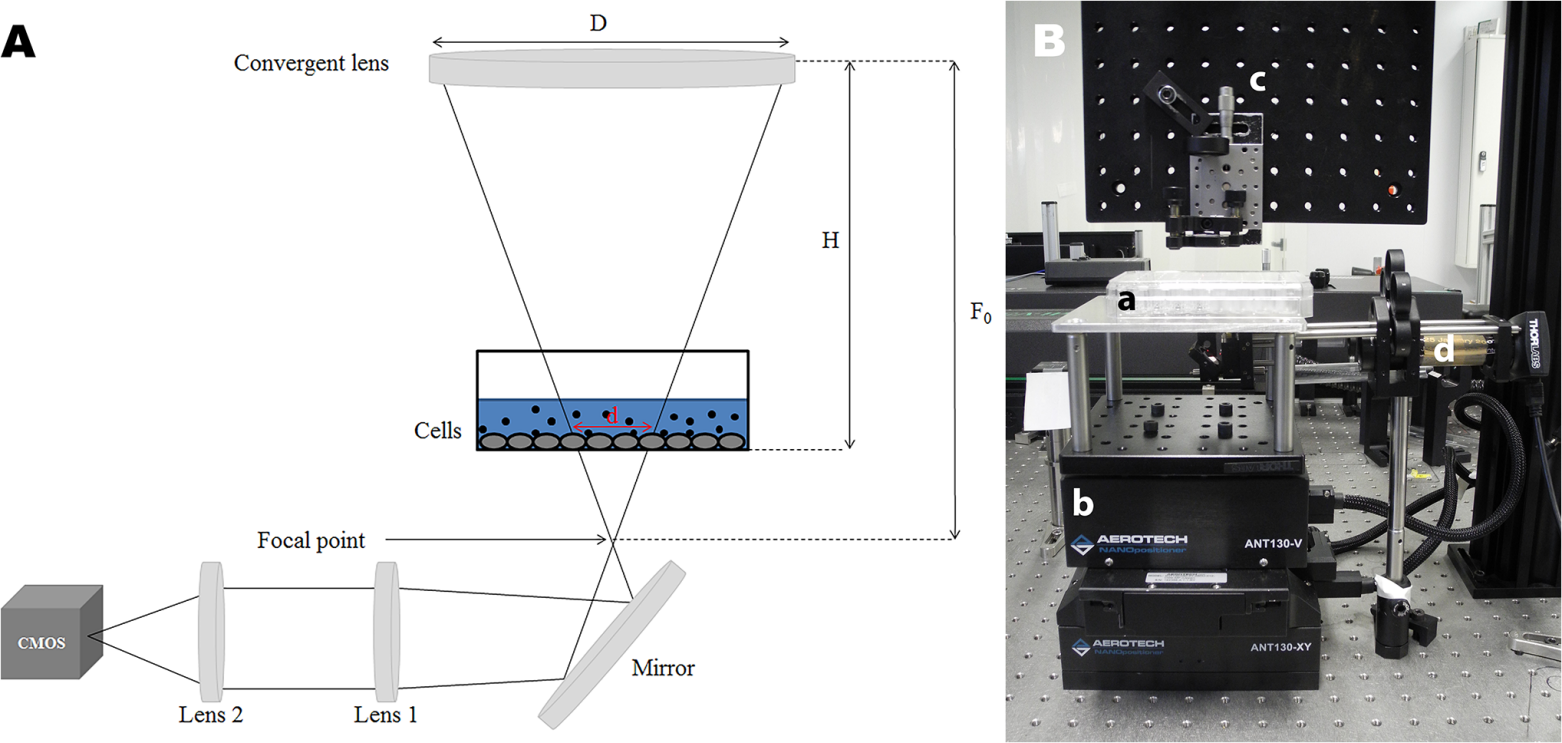
Adjustment of femtosecond laser fluence. (A) Schematic layout. D: Laser beam diameter before focus, H: Distance between lens/cells, d: Effective local spot area, F_0_: Focal distance of the convergent lens; The beam shape was observed in real time in order to verify the actual irradiated area and allow precise calculation of fluence. (B) Experimental settings. a: cell culture plate, b: XYZ motorized stage, c: femtosecond laser path, d: in situ system of beam observation.

Prior to laser irradiation, culture medium was removed and 600μL of the irradiation medium was added. The culture plate was irradiated by a focal spot of a Ti:Sapphire FsL at a wavelength of 800 nm, with a pulse duration of 150 fs and a frequency of 5 kHz. Mean fluence received by CNPs closest to the cells was calculated by dividing the pulse energy delivered by the laser system (in joules) by the focal spot area (in cm²). The FsL irradiation system is shown in Fig 2. The FsL spot diameter was set by the micrometric adjustment of the height of a convergent lens. It was measured precisely using an in situ system of beam observation, composed of two lenses and a camera (CMOS). We successively used two types of FsL irradiation: static and scanning.

Firstly, static irradiation was performed in order to establish the relationships between FsL/CNP/cells interactions and calcein uptake. A 1 mm diameter FsL spot was used to irradiate only a specific zone of the CEC monolayer. Using exposure time of 5 minutes and fluence of 8 mJ/cm^2^, we had previously determined that efficiency of calcein uptake decreased with the 3 CNPs concentrations of 10, 30 and 90 μg/mL (data not shown). The intermediate value of 30 μg/mL was then chosen because it allowed testing a wider range of laser parameters. Three exposure times (3, 5 and 10 min) and three mean fluences (6, 8 and 10 mJ/cm²) were used, as per the initial work of Chakravarty et al. who worked on non-adherent cells [21], and our own preliminary studies.

Secondly, a scanning irradiation was performed in order to irradiate the whole area of the cell monolayer cultivated in a well. An XYZ motorized stage was used to displace the culture plate under the FsL spot by following a spiral trajectory with a velocity of 3.5 mm/s (the same as Baumgart et al. [20]). Preliminary experiments using velocities of 2.0, 3.5 and 5.0 mm/s, all other parameters being fixed, had established a relationship between velocity and efficiency of calcein uptake, with higher uptake with a 2.0 mm/s displacement than with the 2 others (data not shown). The intermediate value was chosen because it allowed testing a wide rage of laser energy; Movements of this stage were controlled with a precision of 250 nm for XY axis. Contrary to static irradiation, the exposure time was considerably reduced due to culture plate displacement, and mean fluences were increased to allow CNP activation. To obtain sufficient fluences (from 38 to 96 mJ/cm²), we used a 500 μm diameter FsL spot.

Immediately after FsL irradiation, the irradiation medium was gently removed, cells were then rinsed with BSS to remove excess calcein and CNPs, and culture medium was renewed. The culture plates were returned to the incubator for 2 hours to reduce cell stress. Three types of controls were performed in the same conditions: (1) CECs alone (no CNPs, calcein or FSL) to determine cell autofluorescence, (2) CECs incubated with CNPs and calcein without FsL to determine spontaneous calcein uptake, and (3) CECs exposed to FsL in presence of calcein but without CNPs to confirm the necessity of laser-CNP interaction. In the latter condition, the absence of CNPs in the medium led to increased laser exposure of CECs because no CNPs absorbed the light, resulting in high cell mortality (data not shown). After having measured, using spectrophotometry, that CNPs alone absorbed half of the energy (data not shown), we divided by two the fluence for this control. Experiments were repeated on three independent cell cultures and for each point at least two wells were considered (2–4).

### Assessment of calcein uptake and mortality

A qualitative method and a quantitative method were used. Two hours after irradiation, cells were first observed with an inverted fluorescence microscope to determine the localization of cells with uptake of calcein (calcein + CECs) and of dead cells, as well as alterations in monolayer organization. After FsL irradiation, a working solution with Propidium Iodide (PI) stock solution (1 mg/mL) at 1:200 in culture medium was prepared and incubated with cells for 15 min at 37°C. Cells were then rinsed and culture medium was renewed. Cultures were observed using a FITC filtre set (ex: 460–500/em: 512–542). Secondly, cells were studied using flow cytometry to quantify delivery efficiency and toxicity. Briefly, cells were harvested from the culture plates using 200 μL trypsin/EDTA 1X for 3 min at 37°C, resuspended in culture medium to neutralize trypsin, centrifuged at 1400 rpm for 10 minutes at 20°C, and diluted at 1x 10^6^ cells/mL in BSS. Cell suspension was assessed using a FACSCalibur flow cytometer in order to quantify the percentage of calcein+ cells (FITC+) and the percentage of PI+ cells (Cy3+). Cell autofluorescence was first determined on cells exposed to neither CNPs nor calcein.

### Subcultivation of cells with calcein uptake

In order to verify whether calcein+ CECs remained viable and kept a normal phenotype, calcein+ CECs were sorted by flow cytometry and subcultured by seeding 3x10^5^ per well in 12-well plates (22.1 mm diameter, 3.8 cm²) until confluence in the medium previously described supplemented by 50 μg/mL gentamycin to prevent any contamination. At confluence, medium was removed and cell monolayer was gently rinsed with BSS, fixed with 4% PFA for staining or methanol 100% for immunostaining for 10 minutes at room temperature and permeabilized with Triton 100X at 0.5% in BSS for 5 minutes at room temperature. The CEC cytoskeletons were then stained with phalloidin at 1:500 for 15 min at room temperature to reveal F-actin, cell nuclei with Hoechst 33342 at 1:200 for 15 min at room temperature to allow determination of endothelial cell density (ECD), and apical tight-junctions were immunolabeled with anti-ZO-1 antibodies at 1:400 for 1 night at 4°C and revealed by AlexaFluor 488 Goat anti-mouse IgG (H+L) antibody secondary antibodies using a protocol that we previously described [22].

### Statistics

Percentages of cells with calcein uptake and of dead cells, quantified by flow cytometry, were described as median (range) and compared with the non-parametric Mann-Whitney test. Analyses were done using IBM SPSS Statistics V.20.

## Results

### Static irradiation

**Fig 3:**
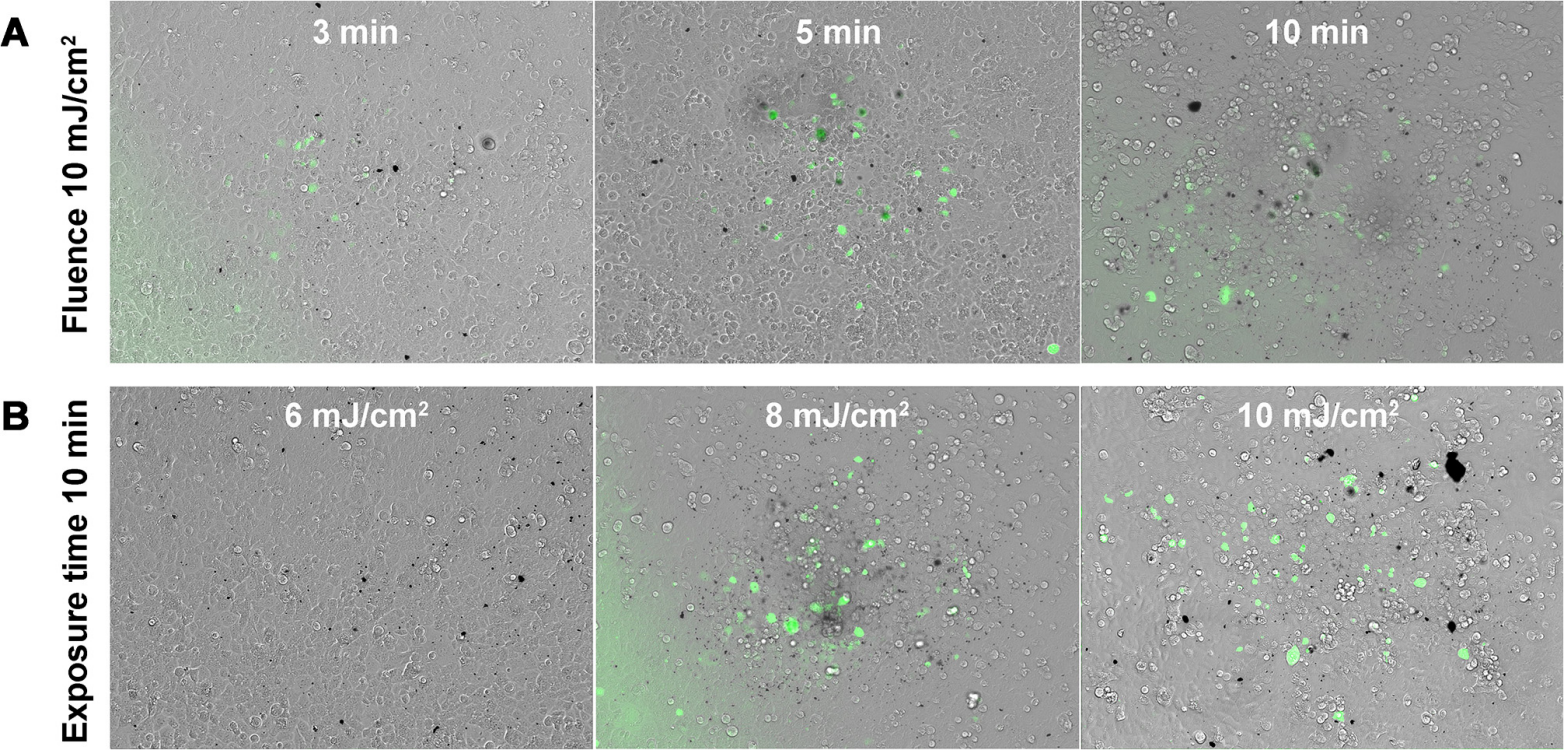
Static laser beam: Effect of fluence and exposure time on calcein delivery into adherent corneal endothelial cells. (A) constant fluence at 10 mJ/cm² and increasing exposure times; (B) constant exposure time at 10 min and increasing fluences. All images were obtained with a x10 objective and merged phase contrast/FITC. Green fluorescence (FITC+) indicated uptake of calcein. Scale bar 200 μm.

No spontaneous calcein uptake was observed in the absence of either FsL irradiation or CNPs. With constant fluence of 10 mJ/cm², calcein+ CECs were visible even for 3 min exposure, but their number seemed to increase with exposure time. The area of the cell culture where uptake of calcein was present was strictly restricted to the area irradiated by the FsL (1 mm in diameter), showing that the reaction was very localized. With a constant exposure of 10 min, calcein+ CECs were visible only from 8 mJ/cm² and increased with fluence (Fig 3). The static irradiation proved the concept of delivery of a small molecule by CNPs activated by a FsL in a monolayer of adherent cells, and of the possibility to modulate efficiency with the laser parameters (fluence and exposure time).

### Scanning irradiation and optimization of laser path

**Fig 4:**
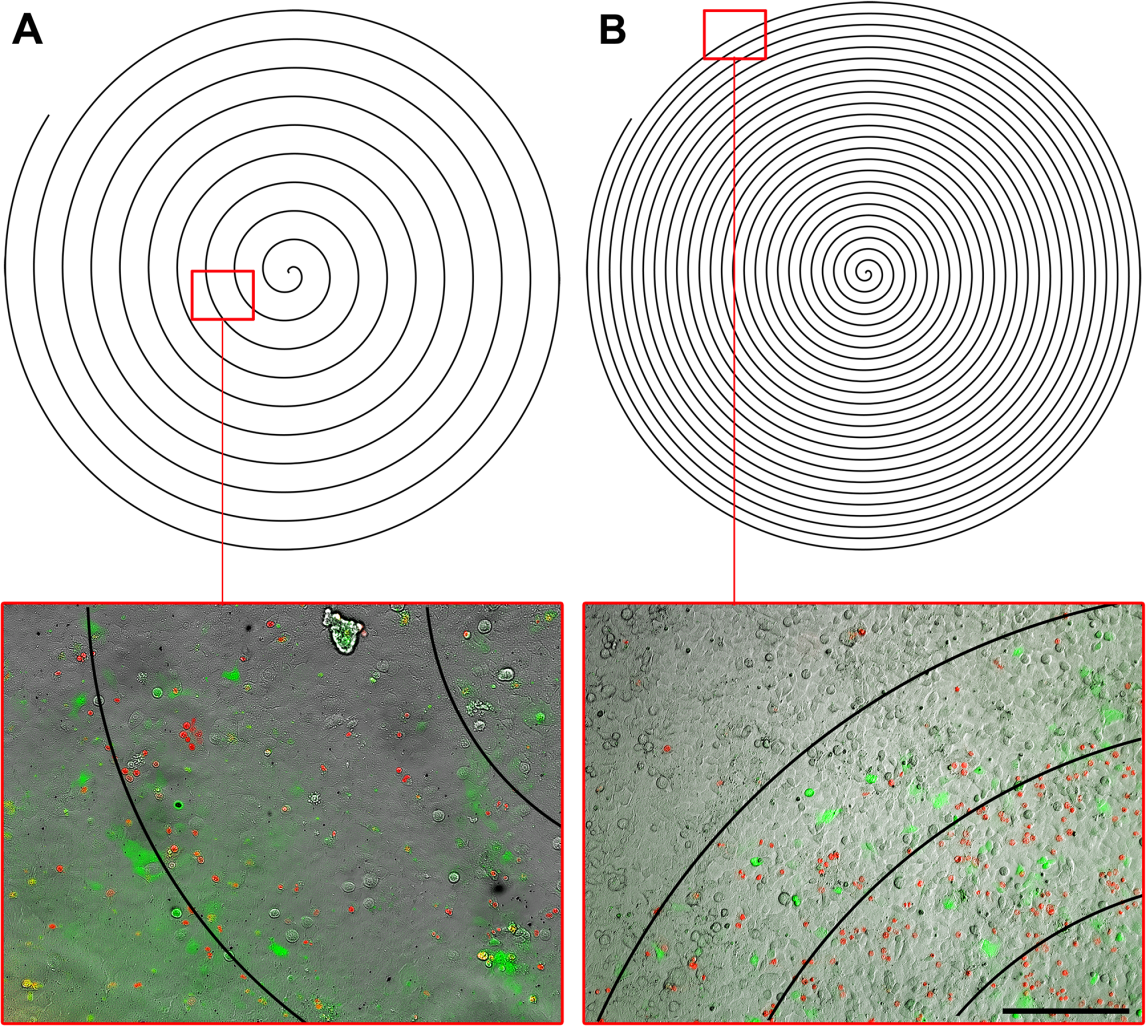
Scanning laser beam: Effect of beam pitch on calcein delivery into adherent corneal endothelial cells. (A) 500 μm pitch; (B) 200 μm pitch; All wells were irradiated at 142 mJ/cm² with a velocity of 3.5 mm/s. Merged phase contrast/Cy3/FITC/overlay representing the laser path. Note that mortality (red PI+ cells) was high in this experiment because fluence was purposely high in order to be sure to obtain uptake of calcein. It was reduced in the subsequent experiments. Green fluorescence (FITC+) indicated uptake of calcein. Scale bar 200 μm.

The whole culture plate area was irradiated using a scanning displacement under the FsL spot with a 15.6 mm diameter Archimedean spiral trajectory. With a spiral space interval (pitch) of 500 μm, corresponding to the FsL spot width, and a velocity of 3.5 mm/sec, the scanning irradiation lasted 2 min but microscopy observations showed that calcein uptake was not homogeneous within the monolayer. The calcein+ CECs seemed more evenly distributed in two 100 μm bands on either side of the FsL spot trajectory. With a space interval readjusted to 200 μm but the same velocity, irradiation lasted 5 min and calcein uptake was more homogenous (Fig 4).

### Scanning irradiation—Quantification of efficiency and toxicity

**Fig 5:**
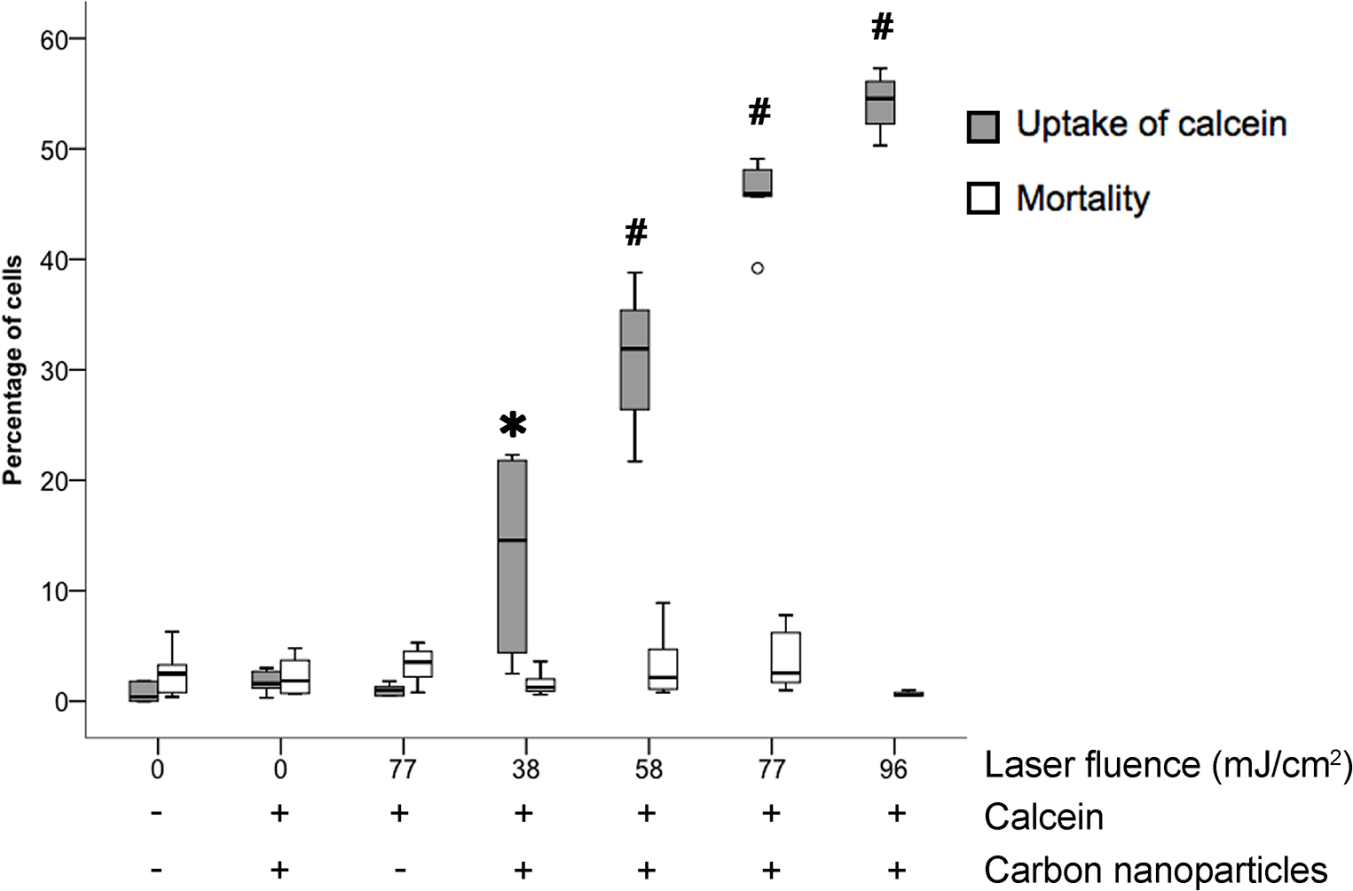
Effect of laser fluence on intracellular uptake of calcein and mortality of corneal endothelial cells. Box plots of the percentage of cells. The thick horizontal lines show the distribution median; boxes, the interquartile range (IQR); and individual circles, outliers. Whiskers show the highest and lowest non-outlying values. A circle is between 1.5 and 3 times the IQR. * P<0.05 for calcein uptake compared to non-irradiated cells. # P<0.05 for calcein uptake compared to the previous point.

Calcein uptake increased significantly and almost linearly with fluence, from 38 mJ/cm² to 96 mJ/cm², while it remained almost nil for the three controls (Fig 5). The best efficiency, median 54.5% (range 50.3–57.3) of calcein+ CECs, was obtained with 96 mJ/cm². Toxicity on adherent cells was not significantly increased by any of the fluences of the scanning laser beam tested. However, we did not measure the rate of cell shedding following FsL irradiation.

### Subcultivation of cells with calcein uptake

**Fig 6:**
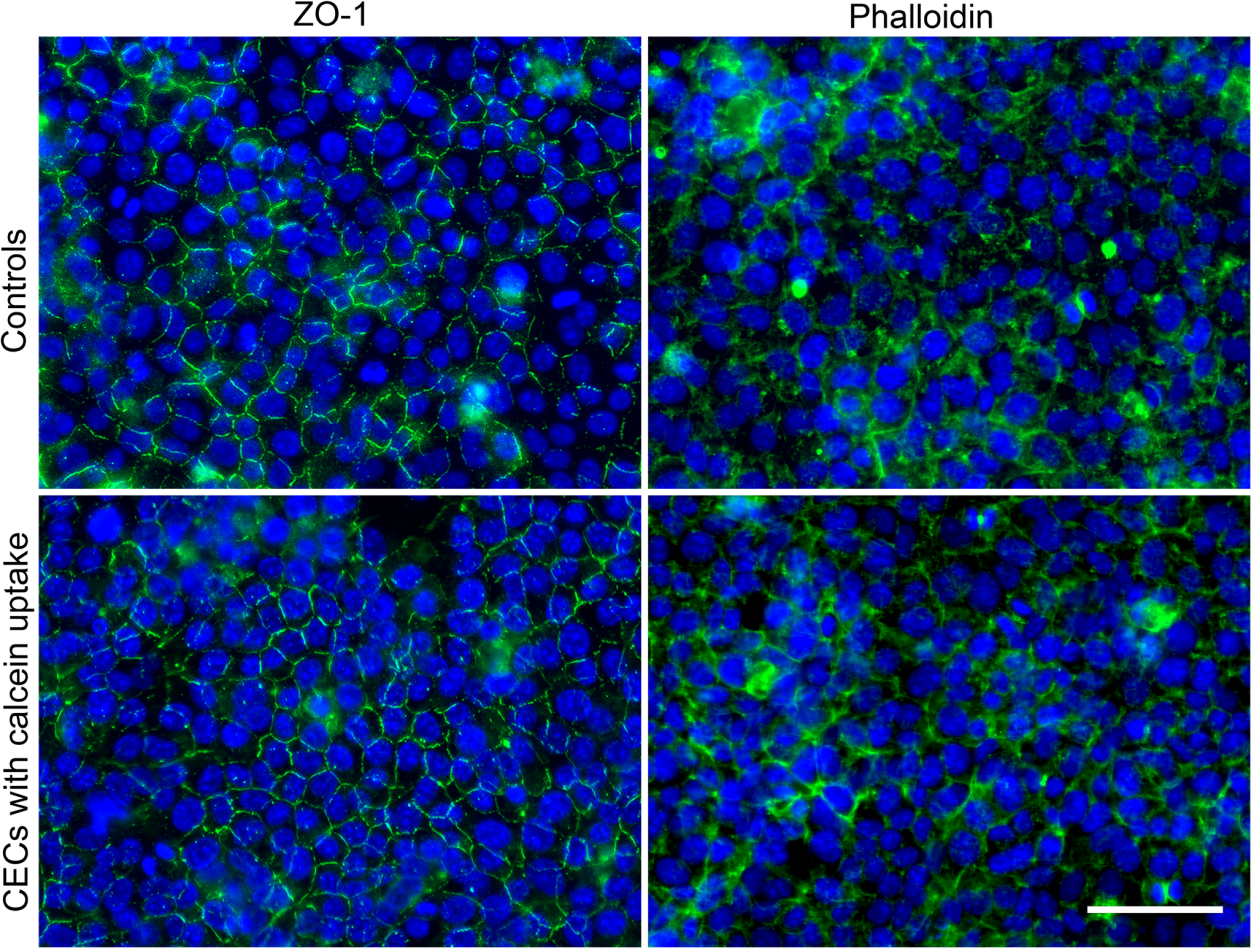
Morphology of calcein positive corneal endothelial cells after subsequent cultivation. ZO-1 and Phalloidin staining on calcein+ cells sorted by flow cytometry and further subcultured for 1 week were compared to control seeded at the same initial density. Nuclei were counterstained with Hoechst 33342. Merged DAPI/FITC. Scale bar 100 μm.

With 3x10^5^ calcein+ CECs seeded in 3.8 cm²-well, confluence was reached after 1 week (triplicate), as for untreated CECs (7+/-1 day for 60 cultures). Twenty-four hours after seeding, cells lost their green fluorescence. At confluence, ECD, phalloidin and ZO-1 staining patterns were fully comparable with unirradiated control CECs (Fig 6).

## Discussion

Permanent or transient alteration of CECs by delivery of specific molecules during corneal storage would help improve the availability, quality and/or outcomes of corneal grafts. The use of an efficient and non-toxic method, easily acceptable by health authorities is essential to envisage clinical applications. In the present study, we reported for the first time the use of CNPs activated by FsL to deliver a small molecule into fully confluent in vitro cultures of CECs, mimicking the native corneal endothelium, which ECD typically varies between 2000 and 3000 cells/mm^2^ in eye banks [23]. For target applications like stimulating CEC proliferation, increasing their resistance to apoptosis and releasing immunomodulators, an efficiency of up to 54% of cells with uptake of a specific molecule and toxicity below 5% can be sufficient.

Delivery of molecules into cells using FsL activated nanoparticles is a recent, highly effective method, described to date on a non-confluent adherent melanoma cell line [20] and on cells of a human prostate cancer line and rat gliosarcoma cells in suspension [21]. The efficiency of this new method, which can compete with viral vectors, led us to first apply the method to a confluent monolayer of tightly packed CECs, before adapting the principle to human corneas. We chose to work with B4G12 cells, one of the three well characterized immortalized lines of CECs, instead of primary CEC cultures, which present huge donor to donor variability in terms of population doubling time, of ECD at confluence, and of morphology, and risk presenting endothelial-mesenchymal transition resulting in major changes in phenotype. We also chose CNPs and not gold nanoparticles because their incubation time was shorter and less expensive, both important characteristics for future routine applications. We found a logical relationship between exposure time, laser fluence, area of cells irradiated with the scanning displacement under the FsL spot, and efficiency of delivery. Like Baumgart et al. [20], we used a scanning irradiation to increase the area of adherent cells exposed. We used irradiation of CECs along a spiral path in order to keep the displacement speed constant and consequently avoid local variation in fluence potentially associated with changes in direction in case of irradiation along parallel lines. Nevertheless, we obtained a lower rate of cells with calcein uptake than previously reported. Indeed, although we probably did not reach maximum efficiency because our FsL settings did not allow us to increase laser power, our maximum rate of 54.5% of cells with calcein uptake is far below the near 100% efficiency reached by both previously described techniques. Baumgart et al. [20] presented efficiency up to 70% and mortality around 80% with fluence of 100–120 mJ/cm². For Chakravarty et al. [21], the best efficiency/toxicity ratio was obtained at a fluence of 10 mJ/cm² for 3 min with 80% efficiency and toxicity under 10%. The main difference with our experiments is the ratio between nanoparticles and the plasma membrane area of each single cell freely exposed to the medium. The entire surface of cells in suspension and approximately half of the surface of non-confluent adherent cells are accessible. In the case of confluent CECs with high ECD, this surface is significantly smaller. Further optimizations remain possible to increase fluence, such as reducing spot diameter and scanning velocity.

Solutions also exist to decrease the mortality triggered by molecule delivery. As plasma membrane damage may be the first cause of toxicity, the use of surfactants that help heal wounds in the phospholipid bilayer after induction of holes by the photoacoustic shockwave could reduce CEC mortality. We previously showed that poloxamers and poloxamines, two macromolecules with surfactant properties, are well tolerated by CECs [24, 25]. Our next step is therefore to assess the extent to which poloxamers and poloxamines could reduce CEC death during molecule delivery by FsL-activated CNPs, while retaining high efficiency.

We must acknowledge several limitations in our study. First, we did not take account of cell detachment inside a well during irradiation and only focused on adherent cells, because it remains difficult to distinguish between whole cells and debris in culture supernatant. However, it is likely that overall mortality is slightly higher than mortality measured only on the adherent fraction. This is also a reason why, as mentioned above, we plan to assess poloxamers and poloxamines in further experiments, especially on whole corneas. Secondly, we assessed the delivery only of a small molecule of 622 Da, and results cannot be extrapolated to bigger ones like proteins or nucleic acids, which require specific experiments in our laboratory. However, delivery of high quantities of small molecules specifically into CECs can in itself be a useful goal because, for instance, rapamycin (approx. 900 Da) or ROCK-inhibitors (approx. 320 Da) have shown potential high interest respectively in corneal endothelial protection against oxidative stress [26] and in wound healing [27]. Finally, in vitro experiments cannot be directly extrapolated to the intact endothelium of stored cornea, although we used the human cell line most similar to the native tissue. To take a step forward, we are determining the parameters to deliver molecules directly into CECs of whole corneas during organ culture. This is particularly challenging because the shape of the cornea and the endothelial folds may induce variation of exposure to the shockwave triggered by the reaction between laser and CNPs.

Notably, cornea is by far the human tissue most exposed to FsL, a fact that could also facilitate the health authorities’ acceptance of this new technique. Delivery of molecules into corneal cells using an FsL could be a new application for ophthalmology FsL, which at present is devoted solely to corneal cutting for refractive surgery and corneal graft. As the FsL is a common device easily available in Western countries and already present in several eye banks for corneal graft preparation, it could be a way to disseminate the new technique while avoiding the need for access to a research FsL.

In conclusion, this study demonstrates that the delivery of small molecules by CNPs activated by a femtosecond laser is efficient and has low toxicity in confluent adherent CECs mimicking the corneal endothelium, and anticipates what could be done on whole human corneas to improve outcomes of corneal transplantations.
